# Supplementary material for: Modelling Skylarks (Alauda arvensis) to Predict Impacts of Changes in Land Management and Policy: Development and Testing of an Agent-Based Model
Source: PLoS One. 2013 Jun 6;8(6):e65803. doi: 10.1371/journal.pone.0065803 (PMC3675089; doi:10.1371/journal.pone.0065803)
Supplement: Supporting Information S4 — The skylark ODdox as a zipped archive. (ZIP) [file pone.0065803.s004.zip › Skylark_ODdox/globals_func.html]

ALMaSS Skylark ODdox: File Members


ALMaSS Skylark ODdox
 2.0


- Main Page
- Related Pages
- Classes
- Files

- File List
- File Members

- All
- Functions
- Variables
- Typedefs
- Enumerations
- Enumerator
- Macros

- b
- c
- d
- f
- g
- i
- l
- m
- p
- r
- s

### - b -

- BeginningOfMonth()
  : ALMaSS\_CmdLine.cpp
- boost\_random010000()
  : ALMaSS\_CmdLine.cpp

### - c -

- CloseDownSim()
  : ALMaSS\_CmdLine.cpp
- CompareDist()
  : skylarks\_all.cpp
- CompPcts()
  : farm.cpp
- CreateLandscape()
  : ALMaSS\_CmdLine.cpp
- CreatePopulationManager()
  : ALMaSS\_CmdLine.cpp

### - d -

- delay()
  : ALMaSS\_CmdLine.cpp
- DumpVegAreaData()
  : ALMaSS\_CmdLine.cpp

### - f -

- FloatToDouble()
  : configurator.cpp
  , ALMaSS\_CmdLine.cpp
  , weather.cpp
  , plants.cpp
  , elements.cpp

### - g -

- g\_chance\_gen01000()
  : ALMaSS\_CmdLine.cpp
- g\_chance\_gen010000()
  : ALMaSS\_CmdLine.cpp
- g\_generator()
  : ALMaSS\_CmdLine.cpp
- g\_rand\_uni()
  : ALMaSS\_CmdLine.cpp
- g\_uni\_dist()
  : ALMaSS\_CmdLine.cpp
- GetProbeInput\_ini()
  : ALMaSS\_CmdLine.cpp

### - i -

- ImpactProbeReport()
  : ALMaSS\_CmdLine.cpp

### - l -

- l\_el\_bug\_percentage\_file()
  : elements.h
- l\_el\_read\_bug\_percentage\_file()
  : elements.h

### - m -

- main()
  : ALMaSS\_CmdLine.cpp

### - p -

- PredProbeReportDay0()
  : ALMaSS\_CmdLine.cpp
- ProbeReport()
  : ALMaSS\_CmdLine.cpp

### - r -

- random()
  : landscape.h
  , ALMaSS\_CmdLine.cpp
- ReadBatchINI()
  : ALMaSS\_CmdLine.cpp
- RunTheSim()
  : ALMaSS\_CmdLine.cpp

### - s -

- SpeciesSpecificActions()
  : ALMaSS\_CmdLine.cpp
- SpeciesSpecificReporting()
  : ALMaSS\_CmdLine.cpp


- Generated on Thu Jan 10 2013 13:15:39 for ALMaSS Skylark ODdox by
   1.8.1.1
